# Repurposed Drugs Celecoxib and Fmoc-L-Leucine Alone and in Combination as Temozolomide-Resistant Antiglioma Agents—Comparative Studies on Normal and Immortalized Cell Lines, and on *C. elegans*

**DOI:** 10.3390/ijms25063226

**Published:** 2024-03-12

**Authors:** Łukasz Uram, Natalia Pieńkowska, Maria Misiorek, Żaneta Szymaszek, Magdalena Twardowska, Michał Siorek, Stanisław Wołowiec

**Affiliations:** 1 Faculty of Chemistry, Rzeszów University of Technology, 6 Powstańcow Warszawy Ave., 35-959 Rzeszów, Poland; luram@prz.edu.pl (Ł.U.); mczygier@prz.edu.pl (M.M.); 163835@stud.prz.edu.pl (Ż.S.); 164811@stud.prz.edu.pl (M.T.); siorek.michal@gmail.com (M.S.); 2 Department of Experimental and Clinical Pharmacology, University of Rzeszów, Kopisto 2a, 35-959 Rzeszów, Poland; n.pienkowska@ur.edu.pl; 3 Medical College, University of Rzeszów, 1a Warzywna Street, 35-310 Rzeszów, Poland

**Keywords:** celecoxib, Fmoc-L-Leucine, glioma U-118 MG, HaCaT immortalized cells, normal human fibroblasts BJ, apoptosis, ATP level, *C. elegans*, toxicity, migration

## Abstract

Glioblastoma multiforme therapy remains a significant challenge since there is a lack of effective treatment for this cancer. As most of the examined gliomas express or overexpress cyclooxygenase-2 (COX-2) and peroxisome proliferator-activated receptors γ (PPARγ), we decided to use these proteins as therapeutic targets. Toxicity, antiproliferative, proapoptotic, and antimigratory activity of COX-2 inhibitor (celecoxib—CXB) and/or PPARγ agonist (Fmoc-L-Leucine—FL) was examined in vitro on temozolomide resistant U-118 MG glioma cell line and comparatively on BJ normal fibroblasts and immortalized HaCaT keratinocytes. The in vivo activity of both agents was studied on *C. elegans* nematode. Both drugs effectively destroyed U-118 MG glioma cells via antiproliferative, pro-apoptotic, and anti-migratory effects in a concentration range 50–100 µM. The mechanism of action of CXB and FL against glioma was COX-2 and PPARγ dependent and resulted in up-regulation of these factors. Unlike reports by other authors, we did not observe the expected synergistic or additive effect of both drugs. Comparative studies on normal BJ fibroblast cells and immortalized HaCaT keratinocytes showed that the tested drugs did not have a selective effect on glioma cells and their mechanism of action differs significantly from that observed in the case of glioma. HaCaTs did not react with concomitant changes in the expression of COX-2 and PPARγ and were resistant to FL. Safety tests of repurposing drugs used in cancer therapy tested on *C. elegans* nematode indicated that CXB, FL, or their mixture at a concentration of up to 100 µM had no significant effect on the entire nematode organism up to 4th day of incubation. After a 7-day treatment, CXB significantly shortened the lifespan of *C. elegans* at 25–400 µM concentration and body length at 50–400 µM concentration.

## 1. Introduction

One of the most common and one of the most malicious CNS tumors is glioblastoma multiforme (GBM), a primary brain cancer that accounts in US for 50.1% of all primary malignant brain and other CNS neoplasms cases with an age-adjusted annual incidence rate of 3.26 per 100,000 population [1]. GBM is astrocytoma lesion classified as grade IV by the WHO and characterized by a high variability of pathological features, including pleomorphic cells with anaplastic nature, high mitotic activity, invasive growth, microvascular hyperplasia, and presence of necrotic centers due to the hypoxic environment [2,3]. Standard treatment involves the safest surgical removal of tumor mass to the greatest extent possible, followed by radiotherapy at a total dose of 60 Gy delivered in 2 Gy doses over 30 sessions across 6 weeks and concomitant daily 75 mg/m^2^ temozolomide (TMZ) administration. Then, six cycles of maintenance TMZ are started at daily dose 150–200 mg/m^2^, which is given for 5 days every 28 days. With this regime, median overall survival (OS) of patients is 16.1 months; however, when the patient’s condition does not allow the introduction of a radio-chemotherapy scheme at the full or any scale, median OS can drop to between 10.6–8.0 and 1.8 months, respectively. Recurrence is almost certain; in those cases, re-surgery and re-irradiation can be considered and treatment with temozolomide, nitrosoureas, and/or bevacizumab can be incorporated [4]. 

Because of the failure of classic antiglioma therapies, there is an urgent need to seek out new treatment solutions, the source of which may be drug repurposing/repositioning [5]. This process is defined as the utilization of existing, clinically approved substances for new applications. This approach is cost-efficient, safer, and capable of reducing time in introducing drugs onto the market when compared with traditional drug development. A one of the compounds that may be considered as a repurposing anticancer drug is celecoxib (CXB). CXB is a non-steroidal, anti-inflammatory drug (NSAID) from the COXIBs class, approved for use in pain and inflammation accompanying the course of osteoarthritis and rheumatoid arthritis, and also off-label in various other musculoskeletal conditions [6]. It has been proven that NSAIDs, including celecoxib, have the ability to prevent and treat certain types of cancer [7]. Celecoxib is considered as a promising anticancer treatment, including as an anti-glioma agent [8,9]. Some papers describe its anti-glioma properties, such as reduction of glioma cell viability by inducing DNA damage and leading to p53-dependent G1 cell-cycle arrest, and induction of autophagy [10] or apoptosis [9]. CXB has been also reported to elevate radiosensitivity and decrease drug resistance in glioma [11,12], including chemoresistance to temozolomide [13]. Results of clinical trials shown that CXB at high dose of 400 mg twice daily indicated some promising effects and was well tolerated [14]. The mechanism of action of CXB is presented as a time-dependent, slowly reversible inhibition of cyclooxygenase-2 (COX-2) [15]. By blocking COX-2, it downregulates the transformation of arachidonic acid to prostaglandins, which are bioactive eicosanoids taking part in pain and inflammatory responses. CXB selectively inhibits human COX-2 (IC_50_ = 0.056 µM) over COX-1 (IC_50_ = 16.52 µM) [16]. Since COX-1 is an isoform constitutively expressed mainly in platelets and gastric mucosa, its function is vital for many physiological responses. Thus, selective inhibitors of COX-2 are characterized by having a less negative impact on platelet aggregation and gastrointestinal mucosa condition. Despite this, they still possess side effects associated with both the cardiovascular system and renal function [16]. 

As inflammation is distinctly connected with cancer progression [17], inhibiting it could induce therapeutic effects. In GBM cells, prostaglandin E2 (PGE2), a major product of COX-2, promotes proliferation, angiogenesis, invasion, and immunosuppression [18]. Moreover, the standard treatment of GBM with radiation and TMZ can induce expression of COX-2 and leading to radiochemoresistance, as overproduction of COX-2/PGE2 in tumor cells is correlated with shorter survival of patients [18,19]. PGE2 may also contribute to activation of the Wnt/β-catenin pathway in GBM cells, which induces their proliferation, invasiveness, treatment resistance, and maintains stemness of GBM stem-like cells [20]. In one of the studies, authors showed that COXIBs can suppress Wnt/β-catenin signaling in GBM cells via COX-2 dependent and COX-2 independent mechanisms, leading to their death [21]. Moreover, there is evidence that the Wnt/β-catenin pathway is involved in negative regulation of peroxisome proliferator-activated receptor γ (PPARγ) signaling [22]. is a member of a group of nuclear receptors, the isoforms of which are expressed at high levels in almost all tissues of the body. PPARγ affects several metabolic functions, including glucose and fatty acid uptake, adipocyte differentiation, and insulin sensitivity [23]. Agonistic actions at the PPARγ receptor in several cancers exert multiple anticancer effects, including reduction of oxidative stress, inflammation, cell proliferation and invasion, and stimulation of apoptosis. Wnt/β-catenin signaling can reduce the expression and activation of PPARγ, whereas PPARγ agonists downregulate β-catenin-dependent expressions of pro-oncogenic target genes. There are several preclinical and clinical studies that show the beneficial effects of PPARγ activation in GBM [24].

Thiazolidinediones (TZDs), a group of compounds that are also PPARγ agonists, are anti-hyperglycemic drugs that can stimulate adipogenesis and cause beneficial reallocation of lipids from visceral storages to subcutaneous adipose tissue [25]. One of the compounds that can activate PPARγ is Fmoc-L-Leucine (FL). It must to be noted that FL exhibits lower potency when compared to rosiglitazone, a full agonist of PPARγ from the TZDs class (Ki = 15.0 µM vs. 0.035 µM, respectively), but has similar maximal efficacy. Therefore, FL can be classified as a selective PPARγ modulator [26] that may result in fewer side effects, as is the case with full agonistic action at PPARγ [23]. In addition, PPARγ agonists have been proven to have the ability to prevent and treat certain types of cancer [27,28]. 

Based on the above data, and considering the lack of effective therapies for 50% of TMZ-treated patients with glioma [29], we have assumed that COX-2 inhibitors and PPARγ agonists could exert amplified anti-glioma effects, since studies report such synergistic tumor-suppressing actions in other cancer types [30,31]. The aim of this study was to evaluate the anti-glioma properties of celecoxib, Fmoc-L-Leucine, or their mixture against showing COX-2 and PPARγ expression, temozolomide-resistant U-118 MG glioma cell line. The activity of these drugs was examined comparatively against normal human fibroblasts (BJ) and immortalized human keratinocytes (HaCaT) with significantly lower level of COX-2 and PPARγ proteins. The obtained results will shed light on the action of Fmoc-L-Leucine and celecoxib administered alone and as a mixture on glioma. In addition, the in vivo toxicity of the tested agents was determined using the nematode *Caenorhabditis elegans*.

## 2. Results and Discussion

### 2.1. Cytotoxicity

The U-118 MG glioma cell line is described as being resistant to TMZ up to 100 µM, or even 250 µM concentration, after 24 and 48 h incubation [32,33,34]. Our cytotoxicity studies confirmed that TMZ had no effect on the viability of these cells after 24 h incubation in the range of 25–200 µM concentration. 

**Figure 1:**
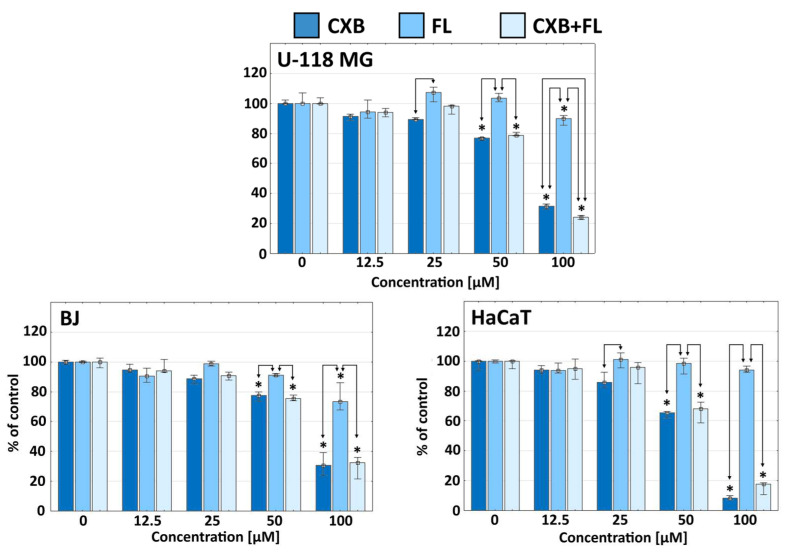
The viability of U-118 MG, BJ, and HaCaT cells after 24 h exposure for CXB, FL, or CXB+FL mixture, estimated with neutral red assay. Results are presented as medians of triplicate assays from three independent experiments, expressed as a % of non-treated controls. The whiskers are lower (25%) and upper (75%) quartile ranges. * *p* < 0.05; Kruskal–Wallis test (against non-treated control), arrows indicate statistical significantly differences between drugs at appropriate concentrations (U Mann–Whitney test).

The obtained data from the NR assay proved that, after 24-h incubation, CXB significantly reduced the viability of U-118 MG glioma cells at a concentration of 50 µM (less than 80%). It had a similar effect on human BJ fibroblasts, and a slightly stronger effect against immortalized HaCaT keratinocytes (viability about 65%) (Figure 1). However, FL had a weaker anti-glioma effect at a 100 µM concentration, but a slightly stronger effect on normal BJ fibroblasts was seen (about 70% viability). 

The authors of several publications postulate an additive or synergistic effect of COX-2 inhibitors and PPARγ agonists in cancer therapy [30]. Our results suggest that CXB and FL administered in a 1:1 ratio did not have an additive or synergistic effect. The activity of the drug mixture always came from CXB; FL, even at the highest concentrations, did not enhance the effect of CXB. Our observations confirm the half maximal inhibitor concentration indexes values (IC_50_) determined with the NR test, the values of which are similar for each cell line. Of all those tested cells, HaCaT cells were the most sensitive on CXB or CXB+FL (Table 1). 

High IC_50_ values for the tested drugs mean that their use in systemic cancer therapy may be limited. Therefore, they should be considered as potential therapeutic agents that may be combined with appropriate carriers and matrices. In the case of glioma, after resection, special matrices and implants are used for local therapy. The matrix material is loaded with a drug and it is possible to significantly increase the life expectancy of patients after resection of glioblastoma and potentially eliminate it completely [36]. The potential solutions include polymeric micelles, polymer nanoparticles, lipid-based drug delivery systems (nanoparticles, micelles, liposomes), hydrogels, and magnetic particles. Additionally, in the case of systemic treatment of glioma, drug carriers can be used, which will allow their penetration through barriers present in the brain, e.g., the blood-brain barrier. More information can be found in the review [37].

### 2.2. Proliferation

**Figure 2:**
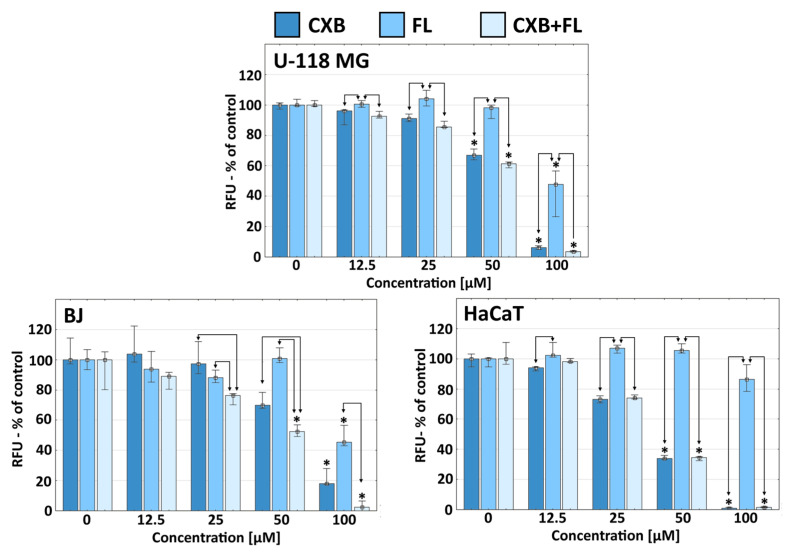
An antiproliferative effect of CXB, FL, or mixture of CXB and FL against U-118 MG, BJ, and HaCaT cells after 72 h incubation. Results are presented as medians and expressed as a % of non-treated controls. The whiskers are lower (25%) and upper (75%) quartile ranges. * *p* < 0.05; Kruskal–Wallis test (against non-treated control), arrows indicate statistical significantly differences between drugs at equal concentrations (U Mann–Whitney test).

To determine whether the effects of the tested drugs occurred by interfering with the cell cycle, a proliferation assay was performed. The antiproliferative effect of celecoxib on cancer, including glioma cells, is widely known [9,10,38]. The antiproliferative effect of CXB against U-118 MG glioma cells was observed at 50 µM concentration, but it was weaker than in the case of immortalized HaCaT cells. The division of glioma and human fibroblast cells, but not HaCaTs cells, was inhibited by FL at a 100 µM concentration (Figure 2).

The antiproliferative effect of an equimolar mixture of CXB and FL was the strongest against HaCaT cells and the weakest against glioma. In this case, the synergism of CXB and FL was observed only in normal human fibroblasts at the highest (50 and 100 µM) concentrations (Figure 2). 

### 2.3. Apoptosis and ATP Level

**Figure 3:**
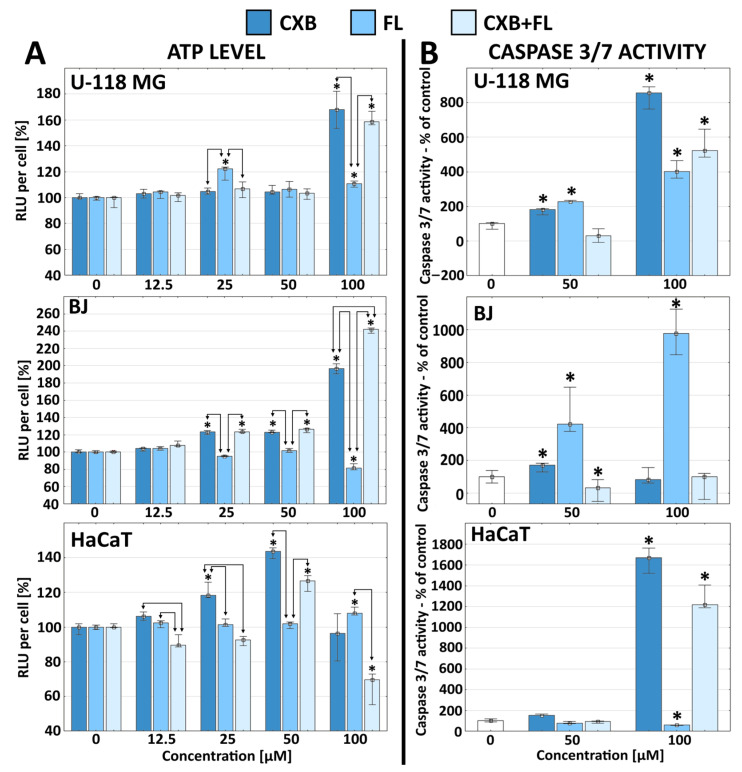
Intracellular ATP level per cell (column **A**) and caspase 3/7 activity per cell (column **B**) in U-118 MG, BJ, and HaCaT cells after 24 h treatment with CXB, FL, or equimolar mixture of both drugs. Results are presented as medians, expressed as a % of non-treated controls. The whiskers are lower (25%) and upper (75%) quartile ranges. * *p* < 0.05; Kruskal–Wallis test (against non-treated control), arrows indicate statistical significantly differences between drugs at equal concentrations (U Mann–Whitney test).

Cell death under the influence of the tested drugs may occur by necrosis, autophagy, pyroptosis, necroptosis, or apoptosis. The latter is a more desirable type of cell death as it does not have negative effects on the surrounding tissues [39]. CXB and PPARγ agonists are known for their pro-apoptotic activity, which may be determined by the activity of executioner caspases: caspase 3 and 7 [40,41]. A study with the Promega apoptosis assay kit showed that caspase 3/7 activity increased in all types of CXB-treated cells, and symptoms of apoptosis were visible with the decrease in cell viability observed in the NR assay from 50 µM (Figure 3B). 

The addition of FL to CXB resulted in a decrease of caspase 3/7 activity only at the 50 µM concentration. It can therefore be concluded that FL was an inhibitory factor weakening the induction of apoptotic changes in these cells at lower (50 µM) concentrations. However, FL used alone induced apoptosis in both U-118 MG glioma and BJ cells at lower concentrations, which were described as toxic in the NR assay (from 50 µM concentration). In HaCaTs, FL reduced the level of caspase 3/7 activity, and these cells were not sensitive to FL.

The decrease of cell viability and the associated apoptotic death process is closely correlated with fluctuations in ATP levels. Cells entering apoptosis usually increase ATP levels to prepare the necessary proteins and mechanisms involved in this process [42,43]. According to other sources, ATP levels may decrease rapidly in apoptotic cells [44]. The data quoted were reflected in the results we obtained. Glioblastoma responded predictably by increasing intracellular ATP levels at toxic 100 µM concentrations of CXB and/or FL; this increase was significantly higher in the case of CXB and the mixture of both drugs when compared to FL (Figure 3A). The pattern of changes in normal BJ fibroblasts was different. The CXB or mixture of drugs at a non-toxic 25 µM concentration caused an increase of ATP levels, but FL at a concentration of 100 µM induced a decrease (Figure 3A). It is particularly surprising that the use of both drugs induced a synergistic effect at the highest concentration, where the ATP level was 240% higher when compared to the control. The profile of energy changes in HaCaT cells was the most unpredictable. CXB increased ATP levels only at 25 and 50 µM concentrations and FL only at 100 µM, while the drug mixture increased ATP levels only at 50 µM but decreased it at the highest concentration (100 µM). 

The data obtained show that different cell types responded dramatically differently to the same agents. There is no simple extrapolation of the increase or decrease in the cell’s energy level depending on the toxicity of the tested drugs and the activity of executive caspases (markers of apoptosis). The explanation for the lack of a clear relationship between the increase in the ATP level and the increase in the level of caspases may be the difference in the sensitivity of cells to the tested compounds, which affects the time needed for the activation of caspases and the occurrence of apoptosis and necrosis. It is also possible that some cells enter the apoptotic death pathway, and some necrotic death results in intermediate resultant values of intracellular ATP levels [43].

### 2.4. Migration

**Figure 4:**
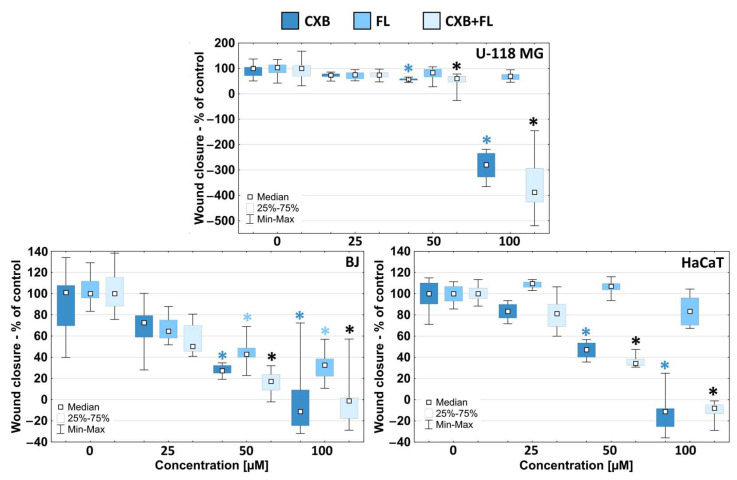
The influence of CXB, FL, or their equimolar mixture on U-118 MG, BJ, and HaCaT cells migration. Results are presented as medians of triplicate assay from three independent experiments, expressed as a % of non-treated controls. * *p* < 0.05; Kruskal–Wallis test (against non-treated control).

The ability of cancer cells to migrate is an important factor in a whole range of changes in cancer development. Migration is an integral step in metastasis, allowing cancer cells to move into surrounding tissues and blood vessels [45]. Therefore, confirming the anti-migratory properties of the tested drugs is a very important issue. In all tested cell lines, a decrease in migration rate was observed alongside an increase in the concentration of the tested drugs. This pattern was consistent with the cytotoxicity profile of the tested drugs. However, U-118 MG glioma cells were more sensitive than both normal and non-tumorigenic immortalized cells. The most sensitive were U-118 MG glioma cells, which migrated slower under the influence of CXB or a mixture of drugs at a 50 µM concentration. It is worth mentioning that the observed effect always came only from CXB. At the highest (100 µM) concentration, the wound area also increased through the detachment of cells adjacent to the scratch area. The wound area increased by about 300–400% (Figure 4).

Normal BJ fibroblast cells showed significant sensitivity to CXB and FL and their mixture from 50 µM concentration upwards. FL acted significantly weaker than CXB or the drug mixture. Fmoc-L-Leucine did not affect the migration of HaCaT cells over the entire range of concentrations used, and CXB or the CXB+FL mixture actively reduced migration from the toxic 50 µM concentration. These data show that FL has the ability to inhibit migration only against normal fibroblasts but not keratinocytes, while CXB against all other cell lines. 

The observed profile of toxicity, proliferation, apoptosis, and migration are consistent with those obtained by others. Celecoxib significantly destroyed A172 glioma cells via the apoptosis pathway after 24 h incubation from 25 µM concentration upward [46]. In a mouse model of malignant glioma (glioma stem cells GSC), celecoxib was toxic (IC_50_ = 60 µM), with overexpression of apoptosis marker (cleaved PARP and caspases) [47]. The latest studies on the effect of celecoxib on four glioma cell lines (U87, U-118 MG, H4, and A172) after 24 h of incubation showed that the IC_50_ values for celecoxib ranged from 100 to 400 µM; U-118 MG cells turned out to be the most resistant (IC_50_ around 400 µM). Cell death occurred by apoptosis [46].

The antiglioma properties of Fmoc-L-Leucine were not tested, but studies on thazolidinediones indicated that PPARγ agonists may induce this effect. Ciglitazone and troglitazone inhibited the growth of U87 glioma tumors and reduced colony formation and migration of tumors at 20 µM concentration. Moreover, the viability of U87 and 8401 glioma cells were significantly reduced from 20 µM concentration after 24 h incubation [21]. In other studies, 17 TZDs derivatives were tested. The mouse C6 and rat GL261 glioblastoma cells, but not primary normal rat’s astrocytes, were sensitive to those compounds at 100 µM concentration. The IC_50_ of the most active was equal (28.51 μM and 54.26 μM, respectively) [48]. These data suggest that we obtained results that corresponded with other data. 

Some studies have shown that TZDs have a selective effect on glioma cells without being toxic to normal, primary astrocytes. Our research clearly excludes lower toxicity towards normal BJ fibroblasts when compared to U-118 MG glioma cells, but HaCaT keratinocytes were not sensitive for FL up to the highest (100 µM) concentration. Such conclusions were also drawn by other authors [47]. 

### 2.5. COX-2 and PPARγ Level

Most brain tumors, including glioma, express or overexpress COX-2, and most human malignant glioma cell lines show constitutively elevated levels of COX-2 [48]. Higher expression levels were observed in glioma tissues of histological grades III–IV compared with those in grade I–II [49]. In our previous studies we have indicated that all tested cell lines (U-118 MG, BJ, and HaCaT) possess constitutive level of COX-2 and PPARγ proteins. In normal human keratinocytes, the COX-2 level was lower and grew in the following order: HaCaT, BJ, and U-118 MG grade IV glioblastoma. Additionally, in glioma cells it was nearly two times higher than in normal and immortalized cells [50,51]. 

**Figure 5:**
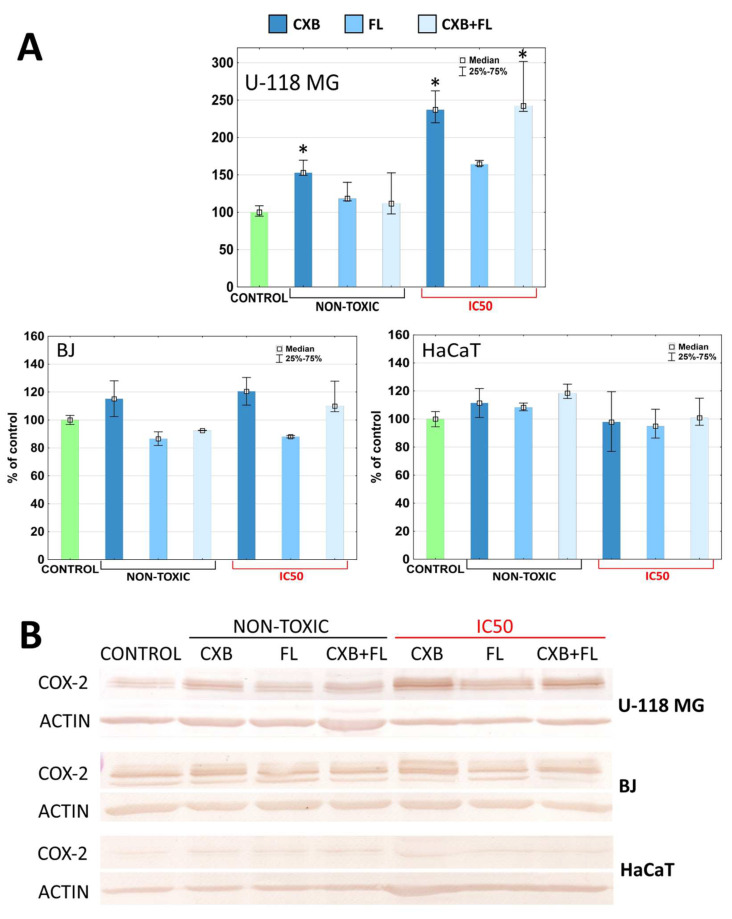
(**A**) The COX-2 level in U-118 MG, BJ, and HaCaT cells after 24 h treatment with CXB, FL, or CXB+FL mixture at non-toxic concentrations and IC_50_. Results are presented as medians expressed as percent of non-treated control. The whiskers are lower (25%) and upper (75%) quartile ranges. Symbol * shows statistically significant difference against control, *p* < 0.05, Kruskal–Wallis test. (**B**) Image of immunoblots.

In this study, levels of COX-2 and PPARγ were examined after incubation with CXB, FL, or an equimolar mixture at non-toxic concentrations (12.5 µM) or IC_50_. In cases where the IC_50_ had a high value, the maximum tested concentration 100 µM was used(see Table 1). Administration of celecoxib, a selective COX-2 inhibitor, at non-toxic concentrations resulted in a significant increase in the amount of COX-2 (by 50%) but only in glioma cells. When IC_50_ = 79 µM, the effect of the drug was even greater and resulted in an increase of COX-2 level by 140%. A mixture of CXB and FL at a toxic concentration (IC_50_ = 73.50 µM) caused an increase in the amount of this protein by 140% (Figure 5A,B). The remaining cell lines did not respond to the tested drugs (Figure 5A,B).

Up-regulation of COX-2 after CXB administration has been observed prior to this study [52]. Higher COX-2 production may be stimulated via a negative feedback loop that was described previously [53,54]. Lombardi et al. observed that temozolomide, one of the three chemotherapy agents available for the treatment of glioma, also causes increase of COX-2 level in glioma cells [55]. This may indicate that the anti-glioma action of CXB possesses a similar mechanism of action. However, down-regulation of COX-2 after 30 µM celecoxib administration in LN229 and LN18 glioma cells was also described [13], as well as no changes in COX-2 expression after CXB administration [56].

**Figure 6:**
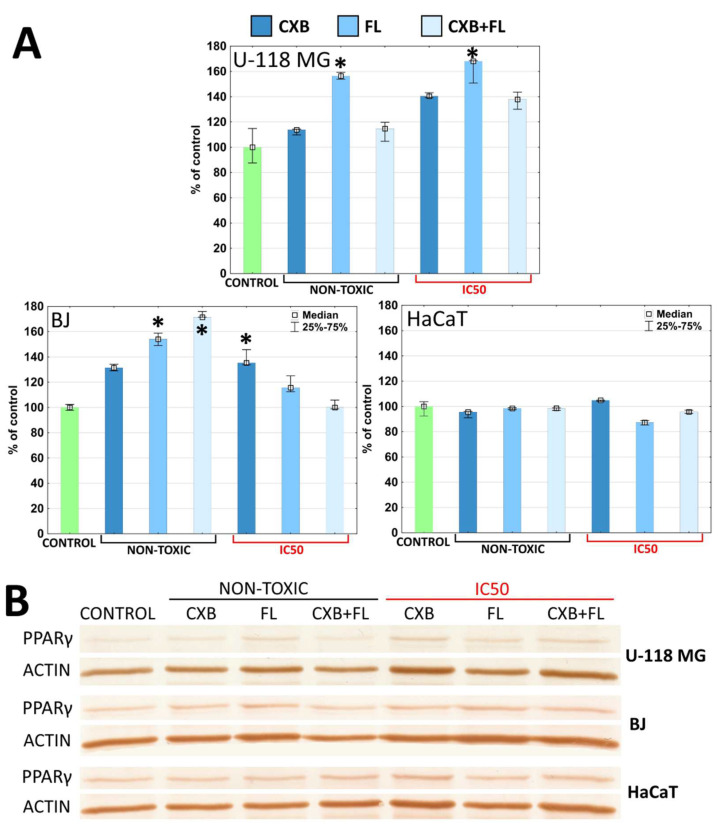
(**A**) Changes of PPARγ level in U-118 MG, HaCaT, BJ, and SCC-15 cells after 24 h incubation with CXB, FL, and CXB+FL mixture at non-toxic concentrations and IC_50_. Results are presented as medians expressed as percent of non-treated control. The whiskers are lower (25%) and upper (75%) quartile ranges. Symbol * shows statistically significant difference against DMSO-treated control, *p* < 0.05, Kruskal–Wallis test. (**B**) Image of immunoblots.

All studied cell lines indicated constitutive expression of PPARγ. PPARγ mRNA was expressed in 95% of the glioma tissue; expression of PPARγ protein was also seen, but in a lower percentage of studied glioma cell lines (for example SK-MG-1 and NB-1) [57]. PPARγ protein levels increased significantly (55%) in both U-118 MG and normal BJ cells after treatment with non-toxic PPARγ agonist FL. At toxic concentrations, FL only caused the same effect in glioma cells (Figure 6A,B). Ching et al. observed concentration dependent elevation of PPARγ levels in both U-87 and U-251 glioma cell lines after 48 h incubation with pioglitazone between a range of 1–100 µM concentration [58]. 

Normal BJ fibroblasts responded with an increase of PPARγ levels after incubation with a mixture of both drugs at non-toxic concentrations (70% increase). Similar results were obtained by Chu et al. in N1-S1 hepatoma cells, where CXB in range of 1 to 100 µM concentration up-regulated PPARγ [59].

Knopfová and Šmarda described complicated connections between COX-2 and PPARγ. They showed that PPARγ ligands may be both COX-2 activators and suppressors, and that COX-2 may regulate PPARγ [30]. The obtained results show that of all the cells tested, only glioma responded predictably to the administered drugs. An increase of COX-2 level occurred after administration of CXB at both tested concentrations. Similarly, the amount of PPARγ increased after incubation with FL at IC_50_ and non-toxic concentrations. There is some evidence that COX-2 inhibitors and PPARγ ligands can synergistically suppress COX-2 and activate PPARγ [30]. No synergism or even an additive effect of the mixture of both drugs on the expression of the tested proteins was observed. In normal fibroblast cells, FL at a non-toxic concentration caused an increase of PPARγ level by 50% and the administration of two drugs simultaneously by 70%. In this case, the synergism of their actions was observed at low, non-toxic concentrations. Only HaCaT cells did not change the expression of COX-2 and PPARγ at any used concentrations of both tested drugs. 

### 2.6. In Vivo Toxicity

A small nematode, *Caenorhabditis elegans*, can be used for testing the toxicity of several compounds and materials, and its handling is simple and inexpensive. Unlike toxicity testing with cell cultures, *C. elegans* toxicity assays provide data concerning a whole animal with intact and metabolically active digestive, reproductive, endocrine, sensory, and neuromuscular systems. As an intermediate between in vitro and mammalian testing, toxin ranking using various *C. elegans* assays has consistently predicted toxicity ranking in mammals [60]. The many similarities between *C. elegans* and mammals allow for cytotoxicity studies of potential drugs, including drugs with anticancer effects [61]. There are studies available that evaluate the relationship between the dose causing toxicity in rats and *C. elegans*. Findings indicate significant parallels, not only regarding dose additivity, but also in terms of potency assessment of substances, between *C. elegans* and the regulatory standard study conducted in rats, which is currently the gold standard for toxicity testing. Research efforts aimed at ranking toxicity in *C. elegans* have consistently demonstrated a correlation with the ranking of rodent oral LD50 toxicity [60,62,63,64,65], although they have some limitations [60]. The fact that adult somatic cells in *C. elegans* are post-mitotic initially suggests that the model might not be ideal for evaluating carcinogenicity, but the cellular mechanisms responsible for DNA replication and repair show high conservation between *C. elegans* and mammals. Additionally, related pathways involved in preventing the spread of carcinogenic mutations, such as apoptosis and cell cycle checkpoints, also exhibit conserved elements [60]. We have chosen *C. elegans* for early studying toxic action of CXB and/or FL on a whole organism as a fully useful model. 

**Figure 7:**
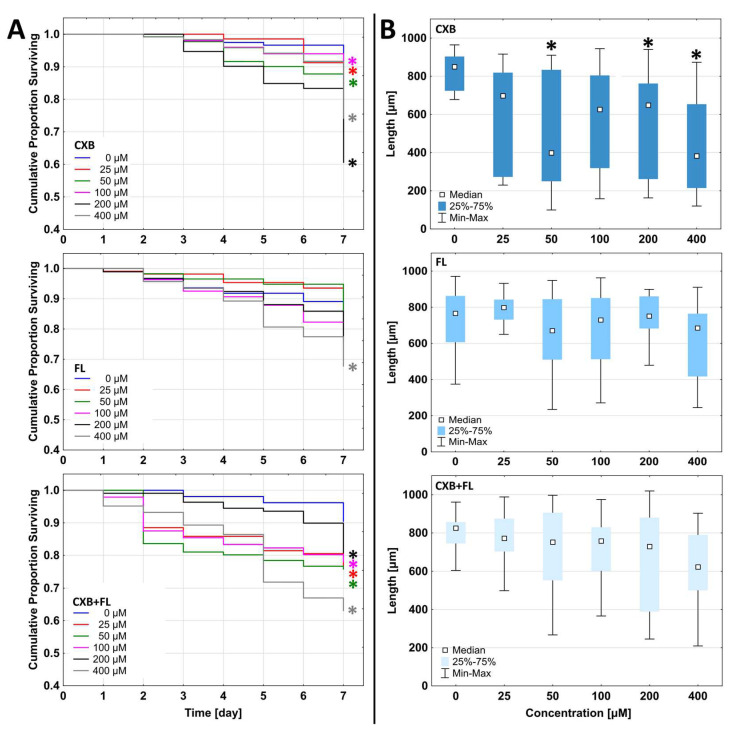
(**A**) The Kaplan–Meier survival curves of *C. elegans* during 7 days of incubation with CXB, FL, or CXB+FL. Results are presented as cumulative proportion surviving. Statistically significant differences against DMSO-treated control obtained in Gehan’s Wilcoxon test are marked with asterisks * (*p* ≤ 0.05) in the colors corresponding to the tested concentrations. (**B**) Changes of body length after 7 days treatment. Statistically significant differences against DMSO-treated control are marked with asterisks * (*p* ≤ 0.05, Kruskal–Wallis test).

**Figure 8:**
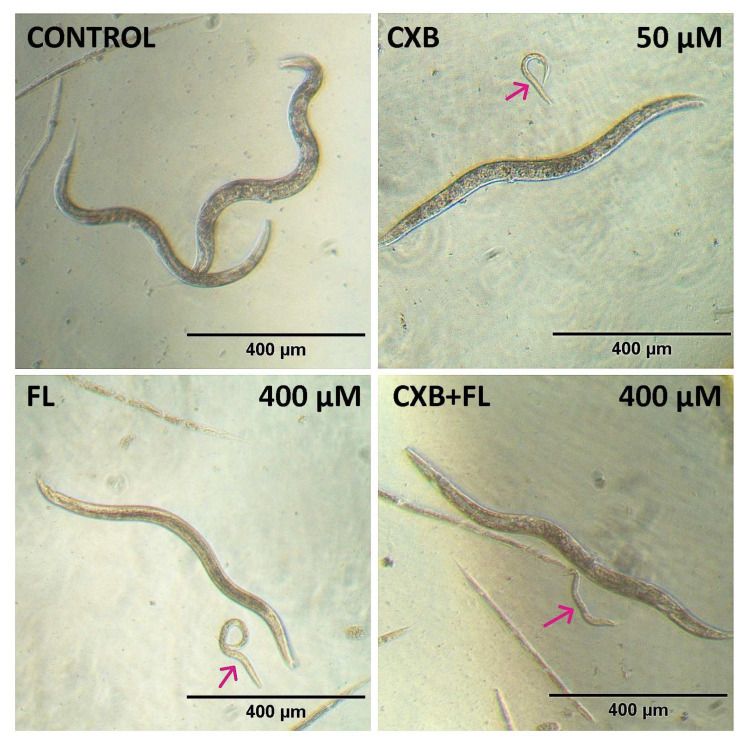
Morphology of *C. elegans* after 7 days incubation with CXB, FL, or CXB+FL. Red arrows show individuals with significantly shorter body length.

Our studies showed that CXB and FL had a negative influence on *C. elegans* in the range of 25–400 µM concentrations after 7 days incubation. The most active was CXB, which was toxic at 25 µM (Figure 7A). Surprisingly, the strongest impact was found at 200 µM concentration (viability lowered to 60%), but not at 400 µM (73% living individuals). During observations, it was noticed that some of the individuals exposed to CXB did not grow and their body length was significantly shorter. At 50 and 400 µM concentrations, these animals were more than twice as short as those in the control group, while those treated with 200 µM concentration of CXB showed a 25% reduction of body length (Figure 7B and Figure 8).

In adult state, wild type *C. elegans* reach approximately 1 mm of length [66], and median of tested nematodes in control was near this value. Body size of *C. elegans* depends on TGFβ and insulin ⁄ insulin-like growth factor (IGF) pathways [67]. Moreover, metabolism, growth, development, longevity, and behavior are connected to nutrient levels by the *C. elegans* insulin/IGF-1 signaling (IIS) pathway [68]. Therefore, in the wild type nematode *C. elegans*, the insulin/IGF-1 signaling (IIS) pathway can function to restrict the body size [69]. There is evidence suggesting that the DAF-2 signal, mediated by the DAF-16 transcription factor, can positively or negatively regulate the expressions of different genes potentially related to nutrient digestion or absorption. These genes likely play significant roles in controlling body size in response to food availability [67]. DAF-16 target genes serve as reporters of the insulin/IGF-1 like signal pathway (IIS), particularly when using *C. elegans* as a model organism to investigate the effects of anti-aging compounds on IIS activity. For instance, CXB, a specific compound, has been found to prolong the lifespan of *C. elegans* by activating DAF-16 [70]. Additionally, CXB at low (10 µM) concentration has been observed to increase the lifespan of animals that have reduced food intake and mitochondrial respiration [71]. Ching et al. conducted a structural-activity analysis that revealed that the anti-aging impact of CXB may not be reliant on its COX-2 inhibitory activity. This is supported by the finding that analogs of CXB without COX-2 inhibitory activity also exhibit a comparable effect on lifespan [71]. We conclude that CXB acts via controlling food uptake and may cause decrease of body length.

FL was significantly less active and showed toxic effects only at the highest (400 µM) concentration. It should be mentioned that by the 6th day of observation, it increased the viability of nematodes at two lowest concentrations: 25 and 50 µM (Figure 7A). A recent study on PPARγ agonist (pioglitazone) showed an increased lifespan (above 10 days of observation) as a result of affecting the insulin/insulin-like signaling (IIS) and reproductive signaling pathways, while also activating pathways associated with dietary restriction (DR) [72]. It allowed us to suppose that FL, as a PPARγ agonist, can also affect IIS pathway and caloric restriction related scales, which can cause body size reduction. In our study, FL did not significantly affect the change in nematode body length, but single nematodes with significantly shortened bodies were observed (Figure 7B and Figure 8). 

*C. elegans* holds promise as a model for analyzing potential mixture effects. Serving as a potentially high-throughput model that combines biological complexity with laboratory simplicity, *C. elegans* has the potential to help identify mixtures of concern for human health [62]. Administration of a mixture of CXB and FL reduced *C. elegans* viability after 7 days of incubation to a greater extent than CXB alone at all concentrations (except 200 µM). Therefore, we conclude that the additive cytotoxicity of these two drugs was observed. 

The IC_50_ of studied drugs and their mixture was much higher than well-known anthelmintic drug, mebendazole (IC_50_ = 4 µM, data from our studies). Therefore, CXB and FL should not be considered as potential anthelmintic drugs when used individually, but may be used as supporting drugs. Obtained results also showed that drugs used at anticancer concentrations (up to 100 µM) will cause rather negligible effects on mammalian organisms such as mice and rats. *C. elegans* also proved to be a useful tool for assessing the toxicity of the tested drugs and their mixtures because the response profile of the nematode was consistent with the response of various types of human cells, including glioma.

## 3. Materials and Methods

### 3.1. Materials

Celecoxib (CXB) was purchased from Fluka (AG, PHR1683, Buchs, Switzerland) and Fmoc-L-Leucine from Sigma-Aldrich (47633, Saint Louis, MO, USA). The powders of CXB and Fmoc-L-Leucine (FL) were dissolved in DMSO (Sigma-Aldrich, Saint Louis, MO, USA) to 100 mM concentration stock solutions. Human glioma cells (U-118 MG), human, normal fibroblasts (BJ), and human squamous carcinoma cells (SCC-15), Eagle’s Minimum Essential Medium (EMEM), Dulbecco’s Modified Eagle’s Medium (DMEM), Dulbecco’s Modified Eagle’s Medium F12 (DMEM-F12), fetal bovine serum (FBS), penicillin, and streptomycin solution were obtained from American Type Culture Collection (ATCC, Manassas, VA, USA). Human immortalized keratinocytes were purchased from Cell Lines Service (Eppelheim, Deutschland, Germany). Trypsin-EDTA solution, phosphate-buffered saline (PBS) with and without magnesium and calcium ions, 0.33% neutral red solution (3-amino-m-dimethylamino-2-methylphenazine hydrochloride), and 0.4% trypan blue solution were provided by Sigma–Aldrich (St Louis, MO, USA). Ethanol and glacial acetic acid were obtained from POCH (Gliwice, Poland). ATP CellTiter-Glo^®^ Luminescent Cell Viability Assay and Caspase-Glo^®^ 3/7 were obtained from Promega (Fitchburg, WI, USA). Fluorescent marker DAPI (4′,6-diamidino-2-phenylindole, dihydrochloride) was provided by Thermo Fischer Scientific (Waltham, MA, USA). 5-Fluoro-20 -deoxy-uridine (FUdR) was purchased from Merck KGaA (Darmstadt, Germany). The bleaching solution was prepared by mixing 5 mL of ddH_2_O, 0.5 mL of 5M KOH, and 1 mL of 4–6% sodium hypochlorite. The other reagents used for nematode culture and synchronization were obtained from Sigma-Aldrich (Saint Louis, MO, USA) or Carl Roth GmbH & Co., KG (Karlsruhe, Germany). Cell culture dishes and dishes for *C. elegans* culture were obtained from Corning Incorporated (Corning, NY, USA) or Nunc (Roskilde, Denmark).

### 3.2. Cell Cultures

The human cell lines—glioblastoma U-118 MG, squamous cell carcinoma SCC-15, normal fibroblasts BJ, and immortalized keratinocytes HaCaT–were used in all experiments. U-118 MG cells (doubling time 35 h) and HaCaT (doubling time 24 h) were cultured in DMEM containing 10% heat-inactivated FBS, 100 U/mL penicillin, and 100 µg/mL streptomycin. SCC-15 cells (doubling time 36 h) were grown in DMEM:F12 supplemented with 10% heat-inactivated FBS, 100 U/mL penicillin,100 µg/mL streptomycin, and 400 ng/mL hydrocortisone. BJ cells (doubling time 45 h) were cultured in EMEM containing 10% heat-inactivated FBS, 100 U/mL penicillin, and 100 µg/mL streptomycin. The cells were incubated at 37 °C and under conditions of 5% CO_2_ and 95% humidity. Growth media were changed every 2–3 days and cells were passaged at 70–80% confluence using 0.25% trypsin- 0.03% EDTA in PBS (calcium and magnesium free). Cell morphology was observed under the Nikon TE2000S Inverted Microscope (Tokyo, Japan) with phase contrast. Cell number and viability were evaluated with Automatic Cell Counter TC20™ (BioRad Laboratories, Hercules, CA, USA), following trypan blue labeling (trypan blue exclusion test). All experiments were performed in triplicate in three independent assays.

### 3.3. Cytotoxicity

To assess the influence of celecoxib (CXB), Fmoc-L-Leucine (FL) or 1:1 mixture on studied cells, one of the most sensitive neutral red assay (NR) was performed [73]. Cells were seeded in flat-bottom 96-well plates at a density of 1 × 10^4^ cells/well (BJ and U-118 MG cells) or 2 × 10^4^ (SCC-15 and HaCaT cells) and allowed to attach for 24 h at 37 °C. Working solutions of drugs (12.5–100 µM) were prepared in culture media, with DMSO concentration adjusted to 0.1% in all samples, which had no significant effect on treated cells. After 24 h exposure to drugs, assay was performed as described [74]. 

### 3.4. Proliferation

Cells were seeded into 96-well, clear microplates at density 4 × 10^3^ cells/well and incubated 24 h at 37 °C to attach. After medium removing, CXB, FL or mixture of both drugs in full culture medium were added at concentrations range from 12.5 to 100 µM (200 µL per well). DMSO concentration was adjusted to 0.1% in all samples. The plates were placed for 72 h in incubator. Then, cells were washed with PBS and fixed in 3.7% formaldehyde solution in PBS for 15 min at RT, again washed with PBS and nuclei were staining with 600 nM DAPI solution in PBS (100 µL/well, 1 h). The fluorescent signal, proportional to the number of cells, was measured in Tecan Infinite M200 PRO Mulitmode Microplate Reader (TECAN Group Ltd., Männedorf, Switzerland) at 360 nm/460 nm. The results were expressed as % of the 0.1% DMSO treated control.

### 3.5. Intracellular ATP Level and Apoptosis

Cells were seeded in 96-well microplates at a density 1 × 10^4^ (BJ and U-118 MG cells) or 2 × 10^4^ (SCC-15 and HaCaT cells). After 24 h incubation at 37 °C, CXB, FL or 1:1 mixture of both drugs were added at range of concentrations from 12.5 to 100 µM (100 µL per well) and plates were incubated for 24 h. 

Intracellular level of ATP was estimated with commercially available bioluminescent CellTiter-Glo^®^ Assay kit from Promega, which was prepared according to manufacturer’s protocol and added to cells (100 μL/well). After 10 min incubation at RT the luminescence was measured using the Infinite M200 PRO Multimode Microplate Reader (TECAN Group Ltd., Männedorf, Switzerland) against blank (wells with the CellTiter-Glo^®^ reagent only). The luminescence signal was proportional to ATP level. At the same time a parallel assay was performed, and cells were labeled with 600 nm DAPI (as described above in proliferation assay). Using calibration curve, the DAPI fluorescence signal was used to estimate the number of cells in each well and to calculate luminescence signal intensity from ATP per one cell. The results were expressed as % of the control (non-treated cells).

As an apoptosis marker, Caspase 3/7 activity was measured with Apo-ONE^®^ Homogenous Caspase-3/7 Assay (Promega). The fluorescence as determinant of caspase-3/7 activity was read at 485/535 nm using Tecan Infinite M200 PRO Multimode Microplate Reader (TECAN Group Ltd., Männedorf, Switzerland) and results were adjusted relatively to the number of cells per well (the number of cells was determined by fluorescence measurement after DAPI staining).

### 3.6. Migration Assay

Cells were seeded in 24-well plates at 1.5 × 10^5^ cells/well in 600 µL culture medium and incubated at 37 °C for 24 h to obtain full confluence. Afterwards, cells were scraped with a sterile 200 µL pipette tips and debris was removed. Culture medium containing 2% FBS with CXB, FL or 1:1 CXB+FL mixture at concentrations between 25–100 µM were added. Medium with lowered 2% FBS was used to inhibit cell division. Plates were left for 12 h (BJ, U-118-MG cells) or 24 h (HaCaT, SCC15 cells) in incubator. Assay was performed as described [75]. The results were expressed as % of scraped area closure estimated as ratio of area measured before and after treatment with drugs.

### 3.7. Western Blot Analysis

BJ, HaCaT, U-118 MG, and SCC-15 cells were cultured for 96 h as described above. Trypsinized cells were lysed with 50 µL per 1 × 10^6^ cells of RIPA buffer (30 min, 4 °C) and centrifuged (13,000 rpm, 4 °C, 15 min.). The protein concentrations in the cell lysates were measured using the Bradford method. The proteins (15 μg of total protein/well) were then separated using 10% SDS-polyacrylamide gels and transferred to PVDF membranes (Immun-Blot PVDF membranes for Protein Blotting, BIO-RAD). After blocking the membranes with a 1% BSA in TBS containing 0.05% Tween-20 (TBS-T) for 2 h at room temperature with constant agitation, the membranes were incubated with a different target primary antibodies (anti-COX-2, NB100-689, NOVUS Biologicals, a dilution of 2000×; anti-PPARgamma, FNab 06660, Fine-Test, a dilution of 1000×; anti-actin, A2066, Sigma-Aldrich, a dilution of 5000×), washed with TBS-T and were incubated with a horseradish peroxidase-conjugated secondary antibody (111-035-689, Jackson ImmunoResearch, a dilution of 10,000×). The blots were then washed with TBS-T and developed with DAB (EMD 1.02924.0001, MilliporeSigma). All immunoblot analyses in this study were performed in two independent experiments. The immunoblots were analyzed with ImageJ 1.49v software (Wayne Rasband, National Institutes of Health, USA).

### 3.8. In Vivo Toxicity

Nematodes wild-type culture (strain N2) was maintained at 20 °C on NGM agar plates with bacteria *Escherichia coli* OP50 as food source. *C. elegans* culture was synchronized by treatment with hypochlorite. Obtained eggs were left in M9 buffer at room temperature to hatch until the following day. The next day L1 worms were placed on NGM plates with *E. coli* OP50 and left at 20 °C until reaching L4 stage (approximately 44 h). L4 worms were transferred to 15 mL falcons by washing NGM plates twice with 5 mL water and centrifuged at 1500 rpm for 3–4 min. Then supernatant was aspirated. Pellet was re-suspended with 5 mL of complete S medium followed by centrifugation [66]. Subsequently the density of nematodes suspension was assessed according to Scanlan et al. [76]. Worms were suspended in complete S medium with *E. coli* OP50 (1:1000), 0.08% cholesterol (5 mg/mL in Et-OH), 1% penicillin-streptomycin, 1% nystatin, and 100 mM FUdR (at final concentration 200 µM) to obtain 20 nematodes in 50 μL. FUdR was added to sterilize nematodes. After that worms were transferred to 96-well plate. The working solutions of tested drugs were prepared in the complete S medium with 0.1% DMSO and placed into a 96-well plate with previously seeded worms. Then, worms were incubated at 21 °C for 7 days. Live (moving and curling) and dead nematodes were counted every day with an inverted microscope with contrast phase (Delta Optical IB-100). Images of treated *C. elegans* were also collected. The assay was performed in triplicates in three independent experiments. To determine the body length of the nematodes, ImageJ 1.49v software (National Institutes of Helath, USA) was used.

### 3.9. Statistical Analysis

To estimate the differences between treated and non-treated control samples, statistical analysis was performed using the non-parametric Kruskal–Wallis test due to the lack of a normal distribution of data in the studied groups (analysis with Shapiro–Wilk test). 

To analyze differences in nematode viability incubated with the respective concentrations of the compounds, the Kaplan–Meier estimator was used. Statistically significant differences between the control and treated groups were determined with Gehan’s Wilcoxon test. Statistically significant differences in body size between the control and treated groups were determined using a Kruskal–Wallis. *p* < 0.05 was considered statistically significant. All analyses and calculations were performed using Statistica 13.3 software (StatSoft, Cracow, Poland).

## 4. Conclusions

Studies on temozolomide-resistant U-118 MG glioma cell line have shown that celecoxib and Fmoc-L-Leucine may be considered as therapeutic agents only as single drugs but not in combination. U-118 MG cells were sensitive from 50 µM concentration and FL from 100 µM, while TMZ remained non-toxic up to a concentration of 200–250 µM. The anti-glioma action of CXB was realized via antiproliferative, proapoptotic, and antimigratory actions, accompanied by an increase in intracellular ATP levels. U-118 MG cells died through COX-2-dependent but not PPARγ-dependent pathways. FL indicated a similar pattern of activity, with a lack of anti-migratory activity and in the PPARγ-dependent pathway. The activity, induced by an equimolar mixture of both drugs, was mainly induced by CXB and was mediated via the COX-2 but not PPARγ up-regulation.

Unfortunately, normal fibroblasts and immortalized keratinocytes reacted similarly. Their death was correlated with PPARγ but not COX-2 overexpression. Only HaCaTs did not respond to FL treatment, with no changes in the levels of the tested proteins. Each of the studied cell lines responded differently under the influence of drugs, which were reflected in difficult-to-predict changes in the level of intracellular ATP. The observations show that the use of CXB or FL in the treatment of glioma may be associated with significant disturbances in the functioning of normal body cells. 

Increased levels of COX-2 and PPARγ expression in glioma cells did not result in increased CXB and FL toxicity in glioma cells when compared to lower expressing BJ and HaCaT cells. Therefore, molecular targets such as COX-2 and PPARγ do not seem to be appropriate therapeutic targets in glioma therapy. However, CXB and FL alone still represent interesting tools in glioma treatment due to their rather low in vivo toxicity and significantly higher activity against temozolomide-resistant glioma cells than TMZ. In vivo studies conducted with model organism, *Caenorhabditis elegans*, confirm the usefulness of that organism in assessing single anti-cancer drug toxicity or potential mixtures thereof.

## Figures and Tables

**Table 1:** The half maximal inhibitory concentration (IC_50_) values determined following 24 h of treatment of U-118 MG, BJ, and HaCaT cells with celecoxib (CXB), Fmoc-L-Leucine (FL), or equimolar mixture of both drugs. The values of IC_50_ were calculated with AAT Bioquest IC_50_ calculator [35]. The lowest IC_50_ values for each drug are underlined.

	IC_50_ [µM]		
	U-118 MG	BJ	HaCaT
CXB	78.56	77.61	59.52
FL	781	400	≫
CXB+FL	73.49	77.82	63.11

## Data Availability

Research data is available from the authors of the publication.
